# Cardiometabolic Risk Factors Among Adults in a Rural Amazonian Peruvian Population

**DOI:** 10.3390/medicina61122206

**Published:** 2025-12-13

**Authors:** Miguel A. Arce-Huamani, Gustavo A. Caceres-Cuellar, Anyela Y. Guevara-Paz, Cleofe R. Lopez-Quispe, Abhely K. Barzola-Blancas, Valeria A. Cespedes-Atto, Catherine G. Acosta-Celis, Katherine Pérez-Acuña, Williams Carrascal-Astola, J. Smith Torres-Roman

**Affiliations:** 1 Public Health, Surveillance and Applied Research, Programa Academico de Medicina Humana, Facultad de Ciencias de la Salud, Universidad Privada Norbert Wiener, Lima 15046, Peru; gustavo2024cc@gmail.com (G.A.C.-C.); yuli94g.p@gmail.com (A.Y.G.-P.); a2021103205@uwiener.edu.pe (C.R.L.-Q.); a2022101130@uwiener.edu.pe (A.K.B.-B.); a2023101399@uwiener.edu.pe (V.A.C.-A.); cathiaccel2@gmail.com (C.G.A.-C.); katherine.perezacuna@unmsm.edu.pe (K.P.-A.); williams.carrasca@uwiener.edu.pe (W.C.-A.); 2 Escuela de Postgrado, Universidad Tecnológica del Perú, Lima 15046, Peru; jstorresroman@gmail.com

**Keywords:** cardiovascular diseases, metabolic syndrome, obesity, abdominal, rural population, Peru, primary health care

## Abstract

*Background and Objectives*: Cardiometabolic diseases are rising in Latin America, yet rural Amazonian populations remain understudied. We aimed to characterize the prevalence and factors associated with a simple composite cardiometabolic risk in rural Amazonian adults. *Materials and Methods*: We conducted an analytical cross-sectional study during community screenings in San Martín, Peru, in 2025, enrolling adults aged ≥ 18 years. The outcome was present when ≥2 biological/anthropometric alterations were identified at the same visit (hypertension, dyslipidemia, hyperglycemia, hyperuricemia, general obesity, abdominal obesity, or elevated waist-to-hip ratio). Behaviors included current tobacco use, alcohol risk (AUDIT), and physical activity (IPAQ). We summarized variables (univariate), compared groups (bivariate: chi-square; Fisher for alcohol), and fitted modified Poisson regression with robust errors to estimate prevalence ratios (PRs); variables with *p* ≤ 0.20 in bivariate analysis entered multivariable models. *Results:* We enrolled 205 adults; 70.2% met the composite outcome. In multivariable models, abdominal obesity (adjusted PR [aPR] 1.70; 95% CI 1.40–2.10), hyperglycemia (1.65; 1.25–2.17), hyperuricemia (1.38; 1.19–1.61), dyslipidemia (1.25; 1.07–1.46), and general obesity (1.21; 1.04–1.40) were independently associated with cardiometabolic risk. Hypertension (1.06; 0.88–1.29) and elevated waist-to-hip ratio (1.20; 0.88–1.63) were not. Physical activity differed crudely but showed no independent association; tobacco and alcohol were not associated. *Conclusions:* In this rural Amazonian population, we observed a high prevalence of composite cardiometabolic risk and found that central adiposity and metabolic derangements, not blood pressure or self-reported behaviors, were the main correlates. Simple measures such as waist circumference, fasting glucose or HbA1c, a basic lipid panel, and serum urate may help flag adults at higher cardiometabolic risk in similar low-resource primary-care settings, but prospective studies are needed to evaluate their predictive value and screening performance.

## 1. Introduction

Cardiometabolic diseases are a leading cause of premature mortality in Latin America and the Caribbean, driven by population aging, urbanization, and rising obesity and diabetes, but their expression is heterogeneous within and between countries [1]. In high-income settings, diabetes prevalence has approximately doubled since 1999–2000, underscoring a sustained expansion of metabolic risk across demographic strata [2]. Emerging data from Latin America suggest similar trajectories, with increasing burdens of hypertension, dyslipidemia, and excess adiposity, including in middle-income countries such as Peru [1]. The COVID-19 pandemic has further shifted population risk by increasing body weight and blood pressure and reducing physical activity among adults [3]. Together, these trends highlight the need for context-specific data on how metabolic and anthropometric abnormalities cluster in underserved populations, particularly in the Amazonian region of Peru.

Cardiometabolic risk rarely arises from a single exposure; instead, combinations of adiposity, hyperglycemia, dyslipidemia, and elevated blood pressure act synergistically to damage the vasculature and target organs [4,5]. Classical frameworks such as the metabolic syndrome and more recent cardiometabolic disease staging systems explicitly capture this clustering and stratify patients according to the number and severity of co-occurring abnormalities [6,7,8]. In parallel, simple composite indices based on routine measurements such as the atherogenic index of plasma, visceral adiposity index, lipid accumulation product, and waist-to-height ratio have been linked to cardiovascular events and all-cause mortality in diverse populations [6]. However, most of these constructs were developed and validated in urban or high-income settings, often require laboratory panels that are not consistently available in community campaigns, and have rarely been examined in rural Amazonian communities [1,9,10]. There is therefore a need for pragmatic, low-cost composite approaches that reflect risk-factor clustering but can be implemented using a limited set of biological and anthropometric measures obtainable during a single field visit.

Rural Amazonian populations in Peru, including those living in the San Martín region, experience a distinct combination of geographic isolation, road and river-dependent transport, nutritional transition, and fragmented primary-care access. Many adults move between traditional subsistence diets and increasing availability of processed foods, while relying on itinerant health campaigns and understaffed health posts for detection and management of chronic conditions. Yet their cardiometabolic profiles remain poorly characterized: most available regional reports describe single risk factors or apply standard metabolic syndrome definitions, offering limited insight into which specific abnormalities most strongly define the local cardiometabolic phenotype [5,7,9]. These gaps constrain the design of screening strategies that are realistic for rural health posts and community campaigns in the Peruvian Amazon.

To address this gap, we developed a simple composite cardiometabolic risk phenotype defined by the presence of at least two biological or anthropometric abnormalities assessed during the same community-based encounter (hypertension, dyslipidemia, hyperglycemia, hyperuricemia, general obesity, abdominal obesity, or elevated waist-to-hip ratio). Unlike formal metabolic syndrome criteria or multistage cardiometabolic classifications, this count-based composite was explicitly tailored to low-resource, rural Amazonian settings and relies on low-cost, routinely obtainable measurements that can be implemented during brief field visits. We hypothesized that, in this context, central adiposity and metabolic derangements would emerge as the main correlates of the composite phenotype, whereas behavioral factors such as self-reported tobacco use, alcohol consumption, and physical activity would show weaker or less consistent associations. Therefore, the objective of the present study was to characterize the prevalence and patterning of this composite cardiometabolic risk phenotype among adults in a rural Amazonian Peruvian population and to identify the specific biological, anthropometric, and behavioral factors most strongly associated with meeting the composite definition.

## 2. Materials and Methods

### 2.1. Study Design and Setting

We conducted an analytical cross-sectional study in rural communities of the Peruvian Amazon (San Martín), Peru. Fieldwork was conducted in 2025 during community-based screening and outreach campaigns organized in collaboration with local health posts. Because recruitment relied on camp-based screening events and door-to-door invitations, the study used a non-probability, convenience sampling strategy, and findings should be interpreted with caution regarding external validity beyond the participating communities. Evaluations and interviews were carried out in community venues (health posts, schools, communal halls) and via door-to-door visits in nearby sectors during the fieldwork period.

### 2.2. Population and Sample

The study population comprised adults aged ≥18 years residing in several rural population centers of the San Martín region who agreed to participate voluntarily during community-based screening and prevention activities. Men and women were invited on site at health fairs and outreach posts or during door-to-door rounds. The included communities were selected in coordination with district health authorities based on program feasibility (existing primary-care posts, community leadership support, and accessibility) and were not designed to be statistically representative of the entire San Martín region. According to local health-post registries, the combined adult population of the participating communities is approximately 1800 individuals; our final sample of 205 adults therefore reflects a sizeable proportion of local residents but should not be extrapolated to the broader region. We excluded individuals with an acute medical condition that prevented a safe evaluation or reliable measurement on the day of assessment, and persons with substantial difficulties understanding questionnaire items despite standardized assistance. Records with incomplete core fields that precluded valid analysis were discarded from inferential procedures.

The sample size was determined a priori for an analytical cross-sectional comparison of proportions between groups exposed and unexposed to cardiometabolic risk factors. In the absence of prior data on a composite cardiometabolic phenotype in this setting, we used regional estimates reporting a 12.4 percentage-point difference in hypertension prevalence between urban and rural adults in San Martín as a sentinel effect size, with a 95% confidence level, 80% power, and 1:1 allocation [11]. This calculation yielded a target sample of 205 adults, which we considered sufficient to ensure adequate statistical sensitivity and precision for associations with the composite outcome, but not to guarantee statistical representativeness at the regional level given the convenience sampling design. Participants were enrolled consecutively until the prespecified target was reached. We ultimately included 205 adults in descriptive analyses; three records had missing values in core variables and were excluded from inferential models. The use of hypertension-based prevalence differences to size a broader composite endpoint is acknowledged as a methodological limitation in the Discussion.

### 2.3. Data Collection

A trained multidisciplinary team (physicians, nurses, and field assistants) collected all data using standardized operating procedures. Sociodemographic information (age, sex, educational level, occupation, and health insurance) was recorded on a structured form. Blood pressure was measured with validated automated oscillometric devices (OMRON HEM-7120, Omron Healthcare Co Ltd., Kyoto, Japan) after ≥5 min of seated rest; two readings were obtained 1–2 min apart in the right arm, and the average was analyzed. Body weight was measured to the nearest 0.1 kg using a digital scale (Seca 874, Seca GmbH & Co. KG, Hamburg, Germany) with participants in light clothing and without shoes. Height was measured to the nearest 0.1 cm using a portable stadiometer (Seca 213, Seca GmbH & Co. KG, Hamburg, Germany). Waist and hip circumferences were obtained with a non-elastic measuring tape (Seca 201, Seca GmbH & Co. KG, Hamburg, Germany); waist circumference was taken at the midpoint between the lowest rib and the iliac crest, and hip circumference at the level of the greatest protuberance of the buttocks.

Fasting capillary or venous blood samples were collected to assess glucose, lipid profile, and uric acid. In field settings, we used a portable point-of-care analyzer (Accutrend Plus system, Roche Diagnostics, Mannheim, Germany) following manufacturer instructions; when on-site processing was not feasible, venous samples were transported to a local hospital laboratory and analyzed with an automated chemistry analyzer (Cobas c111, Roche Diagnostics). Internal quality control procedures required that daily control solutions fall within manufacturer-specified acceptable ranges before participant samples were processed; values outside these ranges triggered recalibration and repeat controls. Lifestyle behaviors were assessed with validated instruments: alcohol use was measured with the Alcohol Use Disorders Identification Test (AUDIT) [12] and physical activity with the short International Physical Activity Questionnaire (IPAQ) [13,14]. Both AUDIT and the short IPAQ have been validated in Spanish-speaking adult populations, including Latin American settings, and we used official Spanish-language versions for data collection.

### 2.4. Variables

The primary outcome was cardiometabolic risk [15], defined as a binary composite coded as present when at least two biological or anthropometric alterations were identified at the same evaluation and coded as absent otherwise. Biological alterations comprised hypertension, dyslipidemia, hyperglycemia, and hyperuricemia [16,17]. Hypertension was defined as systolic blood pressure ≥140 mmHg and/or diastolic blood pressure ≥90 mmHg on examination, or current use of antihypertensive medication. Dyslipidemia was defined as any of the following abnormalities in a fasting lipid profile, or a documented diagnosis or ongoing lipid-lowering therapy: total cholesterol ≥ 200 mg/dL, low-density lipoprotein cholesterol (LDL-C) ≥ 130 mg/dL, high-density lipoprotein cholesterol (HDL-C) < 40 mg/dL in men or <50 mg/dL in women, or triglycerides ≥ 150 mg/dL. Hyperglycemia encompassed prediabetes or diabetes based on fasting plasma glucose thresholds (impaired fasting glucose 100–125 mg/dL; diabetes ≥ 126 mg/dL) or glycated hemoglobin, when available (prediabetes 5.7–6.4%; diabetes ≥ 6.5%), or a documented clinical diagnosis of diabetes. Hyperuricemia was defined as serum uric acid > 7.0 mg/dL in men or >6.0 mg/dL in women, or a documented clinical diagnosis.

Anthropometric alterations comprised general obesity, abdominal obesity, and an elevated waist-to-hip ratio. General obesity was defined as body mass index (BMI) ≥ 30 kg/m^2^ calculated from measured weight (light clothing, no shoes) and height (portable stadiometer) [18]. Abdominal obesity was defined by elevated waist circumference measured at the midpoint between the lowest rib and the iliac crest, using sex-specific cut-offs appropriate for the study population (≥94 cm in men or ≥80 cm in women) [19]. Hip circumference was measured at the point of greatest gluteal protuberance, and the waist-to-hip ratio (WHR) was computed as waist divided by hip circumference; WHR was classified as elevated when >0.90 in men or >0.85 in women. Behavioral variables included current tobacco use (yes/no), alcohol use classified with the Alcohol Use Disorders Identification Test (AUDIT: low risk, at risk, high risk, and dependence), and physical activity categorized with the short International Physical Activity Questionnaire (IPAQ: low/sedentary, moderate, and high). All definitions and thresholds were prespecified, applied uniformly, and derived from same-day measurements, validated questionnaires, or documented diagnoses and therapies.

#### Operational Definitions and Coding

The outcome “cardiometabolic risk” was coded as 1 when at least two of the following domains were present hypertension, dyslipidemia, hyperglycemia, hyperuricemia, general obesity, abdominal obesity, or elevated waist-to-hip ratio and as 0 otherwise. We selected a threshold of ≥2 abnormalities a priori to capture early clustering of cardiometabolic risk factors that would be clinically meaningful in this high-burden, resource-limited setting while maintaining sufficient outcome frequency for stable multivariable modeling. Higher thresholds (e.g., ≥3 abnormalities or strict metabolic syndrome criteria) were considered conceptually but deemed too restrictive for a small rural sample and for a screening-oriented composite. Binary biological and anthropometric exposures (hypertension, dyslipidemia, hyperglycemia, hyperuricemia, general obesity, abdominal obesity, and elevated waist-to-hip ratio) were coded 1 for “present” and 0 for “absent” based on the diagnostic criteria described above. Given the expected correlation among general obesity (BMI), waist circumference, and waist-to-hip ratio, we anticipated some redundancy between anthropometric indices; this collinearity was formally evaluated in multivariable analyses (see Section 2.5) and is considered when interpreting the relative contributions of these measures.

Tobacco use was coded 1 for current smoking and 0 for not smoking. Alcohol use followed AUDIT categorical scoring into low risk (0–7), at risk (8–15), high risk (16–19), and dependence (≥20), and was entered in regression models as a set of indicator (dummy) variables with “low risk” as the reference category. Physical activity followed IPAQ short-form categories (low/sedentary, moderate, high) based on instrument algorithms and was likewise modeled with dummy variables using “low/sedentary” as the reference. Blood pressure was recorded after at least five min of seated rest; two measurements were obtained 1–2 min apart, and the average was analyzed. Anthropometric measurements were taken with calibrated equipment; each circumference was recorded in duplicate, and values outside prespecified tolerances prompted an immediate repeat measure. Laboratory-based biomarkers (fasting glucose, lipid profile, uric acid) were obtained from fasting capillary or venous samples processed with portable analyzers or certified laboratories operating under internal quality control. Records with missing core fields required for the construction of the outcome or key exposures were excluded from inferential analyses; complete-case analysis was used for regression modeling. A detailed summary of all operational definitions and diagnostic thresholds used in the study is provided in Supplementary Table S1.

### 2.5. Data Analysis

We first conducted a univariate analysis to summarize all variables as frequencies and percentages. For the bivariate analysis, we compared the distribution of each exposure by cardiometabolic risk status using chi-square tests; the only exception was alcohol use (AUDIT categories), which we analyzed with Fisher’s exact test due to sparse cell counts. Two-sided *p*-values < 0.05 were considered statistically significant. Variables with a bivariate *p*-value ≤ 0.20 were selected as candidates for the multivariable model. No continuous quantitative variables were analyzed as such; all variables used in descriptive, bivariate, and multivariable analyses were categorical (binary or ordinal), so formal testing of normality for continuous distributions was not required.

In the multivariable analysis, we estimated prevalence ratios (PRs) and 95% confidence intervals using modified Poisson regression with a log link and robust (sandwich) standard errors (vce(robust) option in Stata 18). Categorical exposures (AUDIT and IPAQ categories) were modeled as sets of indicator (dummy) variables with the lowest-risk category as the reference. Multivariable models were fit under a complete-case approach (*n* = 202) because three records had missing values in core variables. We assessed multicollinearity by calculating variance inflation factors (VIFs), considering values > 5 as indicative of problematic collinearity; no covariates exceeded this threshold, and all candidate variables were retained in the final model. We also inspected influence and goodness-of-fit diagnostics as standard practice. Given the individual-level, unclustered sampling frame, alternative cluster-robust or random-effects variance estimators were not required. All analyses were conducted using Stata 18 (StataCorp, College Station, TX, USA).

### 2.6. Ethical Considerations

The study was conducted in accordance with the Declaration of Helsinki and Peruvian regulations for human-subjects research. The protocol, including all field procedures and consent materials, was reviewed and approved by the Research Ethics Committee of Universidad Privada Norbert Wiener (approval No. A0064-2025/CEI-UPW, date: 31 July 2025). All participants provided written informed consent before any study-related procedures. Individual records were de-identified prior to analysis and stored on secure, access-restricted servers.

### 2.7. Data Availability and Use of Generative AI

De-identified datasets and analytic code are available from the corresponding author upon reasonable request and subject to institutional approvals. No generative artificial intelligence tools were used in the design, data collection, statistical analysis, or interpretation of this study.

## 3. Results

During the fieldwork period, community screenings and door-to-door visits were conducted in the selected rural population centers of San Martín. All adults aged ≥18 years who attended the activities or were reached at home and agreed to participate were evaluated. A total of 205 adults completed the assessment and were included in the analyses. As described in the Methods, the use of feasibility-based community selection and convenience sampling means that the study sample should be interpreted as illustrative of adults living in these Amazonian communities rather than statistically representative of the entire San Martín region.

At baseline, 70.2% of participants met the composite definition of cardiometabolic risk, while 29.8% did not. Among biological factors, dyslipidemia (41.9%) and hyperuricemia (23.9%) were common, and hyperglycemia was highly prevalent (72.2%); hypertension affected 18.5%. Regarding anthropometrics, general obesity (36.1%), abdominal obesity (54.6%), and elevated waist-to-hip ratio (78.1%) were frequent. For behavioral factors, current tobacco use was reported by 18.5%, alcohol use fell predominantly in the low-risk AUDIT category (81.5%), and physical activity was mostly low/sedentary (40.9%) or moderate (41.5%), with 17.6% classified as high. Table 1 is intended to describe the overall profile of the cohort, whereas formal statistical comparisons between participants with and without cardiometabolic risk are presented in Table 2.

Chi-square assumptions were satisfied for all comparisons except alcohol use, for which Fisher’s exact test was applied due to sparse cell counts. In bivariate comparisons, cardiometabolic risk clustered with multiple exposures: dyslipidemia (53.5% vs. 14.7% in the no-risk group, *p* < 0.001), hyperglycemia (84.0% vs. 44.3%, *p* < 0.001), and hyperuricemia (31.2% vs. 6.6%, *p* < 0.001). Anthropometric alterations also showed strong contrasts—general obesity (45.8% vs. 13.1%, *p* < 0.001), abdominal obesity (70.1% vs. 18.0%, *p* < 0.001), and elevated waist-to-hip ratio (85.4% vs. 60.7%, *p* < 0.001). Hypertension trended higher among those with risk (21.5% vs. 11.5%, *p* = 0.090). Physical activity differed overall (*p* = 0.035), with a higher proportion of moderate activity in the risk group; tobacco use (*p* = 0.785) and alcohol use (Fisher’s exact, *p* = 0.534) were not associated (Table 2).

In multivariable modified Poisson models restricted to 202 participants with complete data, all listed predictors were entered simultaneously, with biological and anthropometric abnormalities coded as binary indicators (presence vs. absence) and physical-activity categories represented by dummy variables (moderate and high vs. low/sedentary). Cardiometabolic risk remained independently associated with dyslipidemia (adjusted PR [aPR] 1.25; 95% CI 1.07–1.46), hyperglycemia (aPR 1.65; 1.25–2.17), hyperuricemia (aPR 1.38; 1.19–1.61), general obesity (aPR 1.21; 1.04–1.40), and abdominal obesity (aPR 1.70; 1.40–2.10), all statistically significant. Hypertension (aPR 1.06; 0.88–1.29) and elevated waist-to-hip ratio (aPR 1.20; 0.88–1.63) were not independently associated. Relative to low/sedentary physical activity, moderate (aPR 1.02; 0.85–1.22) and high activity (aPR 0.93; 0.76–1.14) showed no independent association with risk (Table 3).

## 4. Discussion

These patterns need to be interpreted in light of the specific characteristics of the study region. Rural Amazonian districts in San Martín are undergoing a rapid nutritional and epidemiologic transition: traditional subsistence farming and physical labor coexist with increasing motorization, sedentary time, and wider availability of processed foods rich in refined carbohydrates, salt, and fats. At the same time, chronic disease detection often depends on itinerant community campaigns and understaffed health posts, with fragmented follow-up and limited access to specialized care. In such environments, long-standing, poorly controlled dysglycemia and dyslipidemia may accumulate silently, while blood pressure remains in earlier stages or fluctuates with acute stressors. This combination offers a plausible explanation for why central adiposity and metabolic markers rather than hypertension or self-reported behavioral factors emerged as the dominant correlates of the composite phenotype in our data.

Our finding that abdominal obesity (waist circumference) is independently associated with higher composite cardiometabolic risk is broadly concordant across diverse settings. In Ecuadorian adults, greater waist circumference and other adiposity indicators were linked to higher blood pressure and HbA1c, reinforcing the centrality of abdominal fat in metabolic dysregulation [20]. Multivariable analyses from India likewise associated hypertension and diabetes with general overweight/obesity and with abdominal or truncal obesity, supporting the primacy of central adiposity beyond overall mass [21]. A regional synthesis from MENA further foregrounds central (abdominal) obesity as a key node co-occurring with dyslipidemia, hypertension, and elevated fasting glucose [22]. Clinical data from Kenya show that most adults with diabetes were overweight/obese and that a high proportion had very high or severe waist circumference, illustrating clustering of central adiposity in high-risk care populations [23]. Even in non-clinical contexts, 21.6% of U.S. college students exhibited high-risk waist circumference, suggesting early accumulation of central fat in younger cohorts [24]. Differences across studies likely reflect sampling frames (community vs. clinic), age structure, ethnic-specific body habits, and outcome definitions (composite risk vs. single components). Collectively, this cross-context consistency strengthens the rationale for waist-based screening in low-resource rural programs, where a simple tape measure can efficiently stratify cardiometabolic risk and inform scalable prevention.

Our result showing an independent association between hyperglycemia and the composite cardiometabolic phenotype is consistent across heterogeneous populations. In Ecuadorian adults, higher adiposity indicators correlated with increased HbA1c, underscoring the tight coupling between glucose dysregulation and adverse metabolic profiles [20]. Clinical data from Kenya further illustrate the glycemic burden, with HbA1c ≥ 7% in 63.4% of men and 53.5% of women attending a diabetes clinic [23]. At the population level, Korea’s fact sheet reports a diabetes prevalence of 15.5% based on fasting plasma glucose ≥ 126 mg/dL, HbA1c ≥ 6.5%, or treatment highlighting how definitional choices capture both chronic glycemia and treated disease [25]. Complementing this, a large Indian analysis estimated diabetes prevalence at 21% and found higher odds among persons with overweight/obesity and with abdominal or truncal obesity, reinforcing shared pathways linking adiposity and hyperglycemia [21]. Differences across studies likely reflect sampling frames (clinic-based vs. community), age structure, and operational definitions (HbA1c vs. fasting glucose vs. treatment), whereas the overall direction of effect remains convergent. Taken together, this alignment supports prioritizing routine glycemic assessment preferably with feasible biomarkers such as HbA1c in rural screening strategies to interrupt progression from dysglycemia to cardiometabolic disease.

Our finding that dyslipidemia independently co-occurs with the composite cardiometabolic phenotype is consistent across both population and clinical settings. In Korea, dyslipidemia affected 40.9% of adults in 2020 based on standard LDL-C, HDL-C, and triglyceride thresholds, underscoring a substantial background burden when components are systematically defined and monitored [25]. Similarly high prevalence was observed in Morocco, where 62.7% of adults met criteria for dyslipidemia in a community survey, indicating widespread lipid abnormalities outside high-income contexts [26]. Indian data further reveal component-level heterogeneity hypertriglyceridemia 15%, high LDL-C 44%, and low HDL-C 75% highlighting that the dyslipidemic pattern can differ by population and risk environment [21]. Complementing these estimates, a synthesis from MENA shows that adults with “normal-weight obesity” have higher odds of dyslipidemia components than normal-weight lean peers, suggesting that adverse lipid profiles can emerge even when overall BMI is not elevated [22]. Apparent differences across studies likely reflect age structure, screening intensity, analytic definitions (full lipid panels vs. composite criteria), and contextual nutrition and activity patterns. Taken together, the convergence of elevated lipid abnormalities across diverse settings supports integrating low-cost lipid assessment with cardiometabolic screening in rural programs, where early identification and targeted counseling could yield outsized prevention benefits.

Our finding that hyperuricemia shows an independent positive association with the composite cardiometabolic phenotype has no direct analog in the provided literature set, as none of the summarized studies reported serum urate or hyperuricemia outcomes. The Ecuadorian analysis prioritized adiposity and glycemic markers linking higher waist circumference to elevated blood pressure and HbA1c without urate metrics [20]. Likewise, the Indian multivariable study focused on general and central obesity in relation to hypertension and diabetes but did not include uric acid [21]. At the synthesis level, MENA evidence emphasized central (abdominal) obesity co-occurring with dyslipidemia, hypertension, and elevated fasting glucose rather than hyperuricemia [22]. This gap limits direct concordance or discordance testing, yet the pathophysiologic plausibility of our signal is reinforced indirectly by the consistent clustering of central adiposity, dysglycemia, and adverse lipids across these sources phenotypes mechanistically intertwined with urate metabolism [20,21,22]. Divergences likely stem from differences in biomarker scope (basic vs. extended panels), laboratory availability in community surveys, and study aims. By explicitly incorporating hyperuricemia in a rural setting, our study adds complementary evidence that may broaden cardiometabolic screening and identify a modifiable target for prevention strategies in low-resource health systems.

Our result that general obesity (BMI) shows an independent though weaker than waist association with the composite cardiometabolic phenotype aligns with population and clinic-based evidence. In Korea, national surveillance documents rising obesity and stratified risks of type 2 diabetes, hypertension, and dyslipidemia across BMI-defined obesity classes, underscoring that higher BMI tracks cardiometabolic burden at the population level [25]. Clinical data from Kenya similarly indicate that most adults attending a diabetes clinic were overweight or obese by BMI, reflecting substantial adiposity among individuals already engaged in care for glycemic disease [23]. In India, overweight (23.5%) and obesity (6.7%) were common, and multivariable models showed higher odds of hypertension and diabetes among those with general overweight/obesity, alongside associations with abdominal/truncal obesity highlighting that overall and central adiposity both mark elevated risk [21]. Differences across these studies likely reflect sampling frames (community surveillance vs. diabetes clinics), age structure, and whether risk is operationalized as single conditions versus composite profiles. Taken together, the concordant direction of effect supports the use of BMI for pragmatic screening, while our weaker signal relative to waist circumference suggests that programs in low-resource rural settings should pair BMI with central adiposity measures to better capture cardiometabolic risk.

In our data, elevated waist-to-hip ratio (WHR) did not retain an independent association with the composite outcome after adjustment despite clear crude differences. This contrasts with high-risk clinical populations, such as a diabetes-clinic sample from Kenya in which high WHR was observed in 53.0% of women and 67.0% of men, illustrating that WHR can be extremely prevalent among individuals with established metabolic disease [23]. Community-based evidence is more heterogeneous: in Morocco, 29.6% of men had increased WHR, whereas central obesity among women was characterized more clearly by waist circumference than by WHR (12.5%), underscoring that the most informative marker of central adiposity can vary by sex and context [26]. Indian analyses have operationalized truncal obesity using WHR cut-offs (≥0.90 in men; ≥0.80 in women) alongside waist circumference criteria, reflecting the concurrent use of both indices in multivariable frameworks [21]. The divergence between our adjusted null findings and these reports is plausibly explained by multicollinearity between WHR, waist circumference, and BMI, random measurement error in hip circumference in community campaigns, ethnic and body-shape differences that alter WHR’s discriminatory power, and clinical enrichment of comparison samples. Taken together, while WHR is prevalent and routinely defined in several settings [21,23,26], our results suggest that, in this rural cohort, waist circumference rather than WHR may be a more practical anthropometric marker for field-based cardiometabolic risk stratification in low-resource primary care.

In our study, hypertension did not retain an independent association with the composite cardiometabolic phenotype after adjustment. This contrasts with community and clinic evidence where blood pressure burden and its links to adiposity are prominent. In Ecuadorian adults, larger waist circumference and other adiposity indicators correlated with higher blood pressure, underscoring the tight coupling between central fat and pressor phenotypes [20]. In a diabetes-clinic cohort from Kenya, hypertension prevalence reached 84.7%, reflecting the concentration of elevated blood pressure within high-risk care populations [23]. At the population level, Korea reported an age-standardized hypertension prevalence of 30.3% in 2021–2022 under explicit diagnostic criteria, highlighting substantial background risk with standardized definitions [25]. Similarly, Indian adults showed a 44% prevalence and higher odds of hypertension among those with general overweight/obesity and with abdominal or truncal obesity, pointing to consistent adiposity–pressure linkages [21]. Our adjusted null may reflect conditioning on correlated metabolic domains (central adiposity, dysglycemia, dyslipidemia), differences in sampling frames (rural community vs. clinic), or operationalization (composite phenotype vs. single-condition outcomes). Even so, the external evidence base affirms that hypertension remains pervasive and metabolically entangled, reinforcing the need for integrated screening and treatment pathways in rural primary care that align blood pressure control with obesity and metabolic risk management.

In our data, physical activity showed crude differences but no independent association after adjustment. External evidence is mixed and context dependent. A MENA meta-analysis found that lower activity was associated with greater odds of the “normal-weight obesity” phenotype versus normal-weight lean, with “good–ideal” physical activity linked to lower odds (OR 0.74, 95% CI 0.60–0.92), suggesting that activity protects against adverse body-composition cardiometabolic profiles [22]. In community surveillance from Morocco, 81.3% of adults reported low physical activity, highlighting a substantial exposure reservoir that could amplify risk when combined with central adiposity and dysglycemia [26]. Among Chinese young adults, 25.8% had inadequate physical activity, indicating early-life lifestyle patterns that may seed later cardiometabolic burden [27]. Our adjusted null could reflect attenuation after conditioning on correlated metabolic mediators (waist, glycemia, lipids), measurement differences in activity assessment across settings, age structure, or limited variability in activity levels within a rural cohort. Overall, while our findings suggest that activity per se was not independently associated after accounting for metabolic factors, external evidence supports integrating physical-activity promotion with waist and glycemic screening to reduce upstream drivers of cardiometabolic risk in resource-constrained primary care.

Our study found no meaningful association for tobacco or alcohol (alcohol tested via Fisher’s exact), a pattern that only partially aligns with external literature. In the MENA synthesis, smoking was not significantly associated with the normal-weight obesity phenotype versus normal-weight lean, echoing our null for tobacco; by contrast, alcohol consumption showed higher odds (OR 1.28, *p* < 0.001), diverging from our alcohol null [22]. Indian data linked alcohol consumption with hypertriglyceridemia, underscoring lipid-specific pathways that our composite outcome may dilute [21]. Background exposure varies widely: current smoking was 9.4% in Moroccan adults [26], whereas among Chinese young adults daily smoking and alcohol intake were 19.6% and 22.9%, respectively [27]; one Kenyan diabetes-clinic study did not capture smoking or alcohol, illustrating reporting gaps [23]. Discrepancies likely reflect outcome construction (single lipid components vs. composite phenotype), cultural consumption patterns, under-reporting in rural settings, and statistical power. Even with a local null, the broader evidence base supports continued alcohol- and tobacco-control strategies alongside metabolic screening to curb cardiometabolic risk in rural health systems.

Our binary composite definition aligns conceptually with risk-factor clustering frameworks, including metabolic syndrome, in that it captures the co-occurrence of central adiposity, dyslipidemia, elevated blood pressure, and dysglycemia, but it differs from several external operationalizations. The Ecuador analysis, for example, referenced harmonized metabolic syndrome criteria central obesity, triglycerides, HDL-C, blood pressure, and glucose to define composite risk, emphasizing component-based clustering based on established diagnostic definitions [20]. The MENA meta-analysis similarly operationalized central obesity, dyslipidemia, hypertension, and fasting glucose to compare body-composition phenotypes rather than a single binary index [22]. By contrast, a Chinese young-adult study treated cardiometabolic disease as a composite of diabetes and/or hypertension and/or dyslipidemia, blending diagnosed conditions with risk factors [27]. The LA BARCA cohort in the U.S. expanded even further, examining cardiometabolic risk factors alongside cardiovascular disease outcomes (coronary heart disease, stroke), a broader endpoint set than a unified “risk” construct [28]. These definitional differences risk-factor clustering vs. established disease, and inclusion or exclusion of CVD events affect prevalence, effect sizes, and cross-study comparability. Our approach, while parsimonious and tailored to rural community screening, should therefore be viewed as a pragmatic risk flag rather than a substitute for formal metabolic syndrome definitions; nonetheless, future harmonization with widely used metabolic-syndrome criteria could facilitate benchmarking and policy translation across regions.

This study advances sparse evidence from rural Amazonian settings by showing that This study advances sparse evidence from rural Amazonian settings and should be interpreted in the context of heterogeneous sampling frames across the literature. Several of the external studies we cite are community-based surveys of adults in the general population, whereas others are clinical cohorts drawn from diabetes or hypertension clinics, where participants have established disease and are under active medical care. Clinic-based samples expectedly show higher prevalence of metabolic abnormalities and more advanced cardiometabolic profiles than community samples and may overrepresent individuals with better access to services. By contrast, our estimates come from adults recruited in community campaigns and door-to-door outreach, with mixed awareness and treatment status. Comparisons with external evidence should therefore focus on the direction and consistency of associations rather than on absolute prevalence levels, and differences in magnitude are likely explained in part by these distinct sampling frames.

Several limitations should be acknowledged. First, the cross-sectional design and single time point of data collection (community campaigns conducted in 2025) preclude causal inference and do not capture temporal changes in risk profiles; our findings should be interpreted as associations rather than effects. Second, camp-based, non-probability recruitment in a subset of rural communities, together with a sample size that was powered analytically using hypertension-based prevalence differences rather than designed for formal regional representativeness, limits the generalizability of estimates beyond the participating localities. The high prevalence of the composite phenotype likely reflects the combined effects of ongoing nutritional transition, occupational patterns, and constrained chronic-care capacity in these rural Amazonian communities, which may differ from other Peruvian regions. Third, single-visit ascertainment with portable analyzers and self-reported behaviors (physical activity, tobacco, alcohol) risks misclassification; under-reporting is particularly plausible for alcohol and tobacco, and hip circumference is especially prone to field measurement variability, which could attenuate waist-to-hip associations. We also lacked detailed data on diet quality and household socioeconomic position (e.g., income or assets), and we captured physical activity at a single time point without accounting for agricultural or seasonal variation that may shape activity patterns in rural Amazonian settings. Residual confounding (e.g., medications, kidney function) is therefore possible, and a composite endpoint may dilute component-specific links (notably alcohol–lipids). Nevertheless, the study has important strengths: standardized anthropometry by trained staff; use of calibrated portable devices with routine quality-control checks; a pre-specified analytic pathway (univariable, bivariable, and multivariable models) using prevalence ratios appropriate for common outcomes; simultaneous evaluation of correlated metabolic domains; and inclusion of hyperuricemia, rarely measured in rural surveys, enhancing construct breadth. Taken together, the internal rigor and programmatic simplicity of the measurements yield actionable priorities for rural primary care and inform resource allocation for cardiometabolic prevention at the health-system level.

## 5. Conclusions

In this rural Amazonian population, we documented a high prevalence of a composite cardiometabolic risk phenotype defined by the presence of at least two biological or anthropometric abnormalities at a single visit, with 70.2% of adults meeting this threshold. Central adiposity, hyperglycemia, dyslipidemia, and hyperuricemia emerged as the main correlates of this composite phenotype, whereas hypertension, elevated waist-to-hip ratio, and self-reported tobacco use, alcohol consumption, and physical activity were not independently associated after adjustment. These findings indicate that, in this setting, simple measurements such as waist circumference and a small biochemical panel (fasting glucose or HbA1c, a basic lipid profile, and serum urate) capture most of the observed clustering of cardiometabolic risk factors. However, the composite index we applied has not yet been validated against hard outcomes, and its prognostic performance and optimal thresholds remain unknown. Before this phenotype is adopted programmatically or used to guide resource allocation, longitudinal studies in rural Amazonian and similar Latin American settings should confirm its predictive value and assess feasibility and acceptability in routine primary care. For now, our results should be interpreted as descriptive and hypothesis-generating, highlighting central adiposity and metabolic disturbances as priority domains for strengthening cardiometabolic prevention in rural health systems.

## Figures and Tables

**Table 1:** Baseline characteristics of the study population.

Variable	*n* (%)
**Cardiometabolic risk**	
No	61 (29.8)
Yes	144 (70.2)
**Biological factors**	
Hypertension	
No	167 (81.5)
Yes	38 (18.5)
Dyslipidemia	
No	119 (58.1)
Yes	86 (41.9)
Hyperglycemia	
No	57 (27.8)
Yes	148 (72.2)
Hyperuricemia	
No	156 (76.1)
Yes	49 (23.9)
**Anthropometric factors**	
General obesity (BMI 30 or more kg/m^2^)	
No	131 (63.9)
Yes	74 (36.1)
Abdominal obesity (waist circumference)	
No	93 (45.4)
Yes	112 (54.6)
Elevated waist-to-hip ratio	
No	45 (21.9)
Yes	160 (78.1)
**Behavioral factors**	
Tobacco use	
No	167 (81.5)
Yes	38 (18.5)
Alcohol use	
Low risk	167 (81.5)
At risk	25 (12.2)
High risk	5 (2.4)
Dependence	8 (3.9)
Physical activity	
Low/sedentary	84 (40.9)
Moderate	85 (41.5)
High	36 (17.6)

Notes: Data are presented as *n* (%). This table provides descriptive characteristics of the total cohort; statistical comparisons by cardiometabolic risk status are reported in Table 2. BMI, body mass index.

**Table 2:** Bivariate associations between cardiometabolic risk and risk factors.

Variable	Cardiometabolic Risk		*p* Value
	No *n* = 61	Yes *n* = 144	
	*n* (%)	*n* (%)	
**Biological factors**			
Hypertension			0.090
No	54 (88.5)	113 (78.5)	
Yes	7 (11.5)	31 (21.5)	
Dyslipidemia			<0.001
No	52 (85.3)	67 (46.5)	
Yes	9 (14.7)	77 (53.5)	
Hyperglycemia			<0.001
No	34 (55.7)	23 (16.0)	
Yes	27 (44.3)	121 (84.0)	
Hyperuricemia			<0.001
No	57 (93.4)	99 (68.8)	
Yes	4 (6.6)	45 (31.2)	
**Anthropometric factors**			
General obesity (BMI 30 or more kg/m^2^)			<0.001
No	53 (86.9)	78 (54.2)	
Yes	8 (13.1)	66 (45.8)	
Abdominal obesity (waist circumference)			<0.001
No	50 (82.0)	43 (29.9)	
Yes	11 (18.0)	101 (70.1)	
Elevated waist-to-hip ratio			<0.001
No	24 (39.3)	21 (14.6)	
Yes	37 (60.7)	123 (85.4)	
**Behavioral factors**			
Tobacco use			0.785
No	49 (80.3)	118 (81.9)	
Yes	12 (19.7)	26 (18.1)	
Alcohol use			0.534
Low risk	50 (82.0)	117 (81.2)	
At risk	9 (14.7)	16 (11.1)	
High risk	0 (0.0)	5 (3.5)	
Dependence	2 (3.3)	6 (4.2)	
Physical activity			0.035
Low/sedentary	33 (54.1)	51 (35.4)	
Moderate	18 (29.5)	67 (46.5)	
High	10 (16.4)	26 (18.1)	

Notes: Data are presented as *n* (%). *p*-values are from chi-square tests, except for alcohol use (AUDIT categories), which was analyzed with Fisher’s exact test due to sparse cell counts. BMI, body mass index; AUDIT, Alcohol Use Disorders Identification Test.

**Table 3:** Crude and adjusted prevalence ratios for cardiometabolic risk.

Variables	Unadjusted PR (95% CI)	*p*-Value	Adjusted PR (95% CI)	*p*-Value
**Biological factors**				
** Hypertension**				
** No**	Reference		Reference	
** Yes**	1.21 (1.00–1.45)	0.047	1.06 (0.88–1.29)	0.501
** Dyslipidemia**				
** No**	Reference		Reference	
** Yes**	1.59 (1.34–1.90)	<0.001	1.25 (1.07–1.46)	0.006
** Hyperglycemia**				
** No**	Reference		Reference	
** Yes**	2.03 (1.46–2.80)	<0.001	1.65 (1.25–2.17)	<0.001
** Hyperuricemia**				
** No**	Reference		Reference	
** Yes**	1.45 (1.25–1.67)	<0.001	1.38 (1.19–1.61)	<0.001
**Anthropometric factors**				
** General obesity (BMI 30 or more kg/m^2^)**				
** No**	Reference		Reference	
** Yes**	1.50 (1.27–1.76)	<0.001	1.21 (1.04–1.40)	0.013
** Abdominal obesity (waist circumference)**				
** No**	Reference		Reference	
** Yes**	1.95 (1.55–2.45)	<0.001	1.70 (1.40–2.10)	<0.001
** Elevated waist-to-hip ratio**				
** No**	Reference		Reference	
** Yes**	1.65 (1.19–2.28)	0.003	1.20 (0.88–1.63)	0.245
**Behavioral factors**				
** Physical activity**				
** Low/sedentary**	Reference		Reference	
** Moderate**	1.29 (1.06–1.59)	0.012	1.02 (0.85–1.22)	0.819
** High**	1.19 (0.91–1.55)	0.202	0.93 (0.76–1.14)	0.495

Notes: PR, prevalence ratio; CI, confidence interval; BMI, body mass index. The adjusted model simultaneously included all variables listed and was restricted to 202 participants with complete data on all predictors. For binary predictors, the reference category was absence of the condition (no hypertension, no dyslipidemia, no hyperglycemia, no hyperuricemia, no general obesity, no abdominal obesity, and no elevated waist-to-hip ratio). For physical activity, low/sedentary was the reference category, with moderate and high activity entered as dummy variables. Statistically significant results (*p* < 0.05).

## Data Availability

De-identified participant-level data and annotated analysis code are available from the corresponding author upon reasonable request and subject to approval by the Universidad Privada Norbert Wiener Research Ethics Committee. To protect participant privacy and small-community confidentiality in the rural Amazonian setting, datasets are not publicly posted. No new data were created beyond these de-identified files.

## Supplementary Materials

The following supporting information can be downloaded at: https://www.mdpi.com/article/10.3390/medicina61122206/s1, Table S1: Operational Definitions and Thresholds for Cardiometabolic, Anthropometric, and Behavioral Variables Assessed in the Study.

## Abbreviations

The following abbreviations are used in this manuscript:

aPR	Adjusted Prevalence Ratio
BMI	Body Mass Index
IC95%	95% Confidence Interval (Intervalo de Confianza al 95%)
RP	Prevalence Ratio
CI	Confidence Interval
