# Supplementary material for: Cardiometabolic Risk Factors Among Adults in a Rural Amazonian Peruvian Population
Source: Medicina (Kaunas). 2025 Dec 13;61(12):2206. doi: 10.3390/medicina61122206 (PMC12734852; doi:10.3390/medicina61122206)
Supplement: Supplementary file 1 [file medicina-61-02206-s001.zip › medicina-4011667-supplementary.pdf]

Supplementary Table S1. Operational Definitions and Thresholds for Cardiometabolic, Anthropometric, and Behavioral Variables Assessed in the Study

Domain	Variable	Operational definition	Threshold / coding
**Biological**	Hypertension	Elevated blood pressure or treated hypertension	SBP ≥140 mmHg and/or DBP ≥90 mmHg, or antihypertensive treatment
**Biological**	Dyslipidemia	Abnormal fasting lipid profile or treated dyslipidemia	TC ≥200 mg/dL, or LDL-C ≥130 mg/dL, or HDL-C <40 mg/dL (men) / <50 mg/dL (women), or TG ≥150 mg/dL, or lipid-lowering therapy
**Biological**	Hyperglycemia	Prediabetes or diabetes	FPG 100–125 mg/dL (prediabetes) or ≥126 mg/dL (diabetes), and/or HbA1c 5.7–6.4% (prediabetes) or ≥6.5% (diabetes), or diagnosis
**Biological**	Hyperuricemia	Elevated serum uric acid	>7.0 mg/dL (men) or >6.0 mg/dL (women), or diagnosis
**Anthropometric**	General obesity	Excess overall adiposity	BMI ≥30 kg/m²
**Anthropometric**	Abdominal obesity	Central adiposity by waist circumference	WC ≥94 cm (men) or ≥80 cm (women)
**Anthropometric**	Elevated WHR	Central adiposity by waist-to-hip ratio	WHR >0.90 (men) or >0.85 (women)
**Behavioral**	Tobacco use	Current smoking	Yes / No (current smoker vs. non-smoker)
**Behavioral**	Alcohol use	Alcohol consumption and risk	AUDIT categories: low risk (0–7), at risk (8–15), high risk (16–19), dependence (≥20)
**Behavioral**	Physical activity	Self-reported activity level	IPAQ categories: low/sedentary, moderate, high
